# Epstein-Barr Virus-Associated Smooth Muscle Tumor in the Liver Post Kidney Transplant: A Case Report

**DOI:** 10.7759/cureus.74441

**Published:** 2024-11-25

**Authors:** Zeshan R Ali, Burak Sevim, Ramy Shoela

**Affiliations:** 1 Radiology, Saint Louis University School of Medicine, St. Louis, USA

**Keywords:** ebv-smt, immunosuppresion, large liver mass, rare tumors, smooth muscle tumor, transplant kidney

## Abstract

Epstein-Barr virus (EBV) is one of the most common causes of infection from the herpes virus family which also possesses oncogenic potential. EBV-associated smooth muscle tumors (EBV-SMT) are often found in the CNS but here we present the case of a 50-year-old woman with EBV-SMT in the liver. This patient had a kidney transplant in 2009 and had been undergoing immunosuppressive therapy to support her transplant. Subsequent imaging found a liver mass which was not seen on previous imaging. The biopsy revealed an EBV-SMT. The exact pathophysiology of EBV-SMT is not clear though it is believed to involve the reactivation of latent infection through mTOR pathways. Treatment of such masses includes reducing immunomodulating pharmacotherapy though no established management guidelines exist.

## Introduction

Epstein-Barr virus (EBV) is one of the most common human pathogens from the herpesvirus family with oncogenic potentials [1]. While they can occur almost anywhere in the body, the most common site of involvement is the central nervous system (CNS) [2,3]. In addition, the GI tract, liver, lung, pharynx, larynx, and skin are the areas where they are frequently seen [2,3]. Although these tumors are quite rare, they are frequently seen in times when the immune system is suppressed, especially in the HIV/AIDS patient population, as well as in immunodeficiency syndromes (such as common variable immune deficiency syndrome) and post-transplant patients [1,2,4]. At the same time, EBV can cause the proliferation of smooth muscles, causing a condition called EBV-associated smooth muscle tumor (SMT) (EBV-SMT) [1,2,5].

The incidence of SMT is not well known. Stubbins et al. found that the incidence of post-transplant SMT in their institute was 0.7 per 1000 patient years [6]. In addition, the median time at diagnosis of EBV-SMT after kidney transplantation is variable. In 2020, Tardieu et al. reviewed 59 post-transplantation SMT cases between 1996 and 2019 [7]. The median time of diagnosis was 74.6 months and 31 were found in the liver. Tan et al. found the median time of diagnosis after transplant to be 9.4 years in their study in 2013 [8]. This rarity poses challenges for diagnosis and management, necessitating further research into its etiology and optimal treatment approaches. 

Here, we present a case in which an EBV-SMT was found in the liver of a 50-year-old female patient who underwent kidney transplantation almost 15 years ago.

## Case presentation

**Figure 1:**
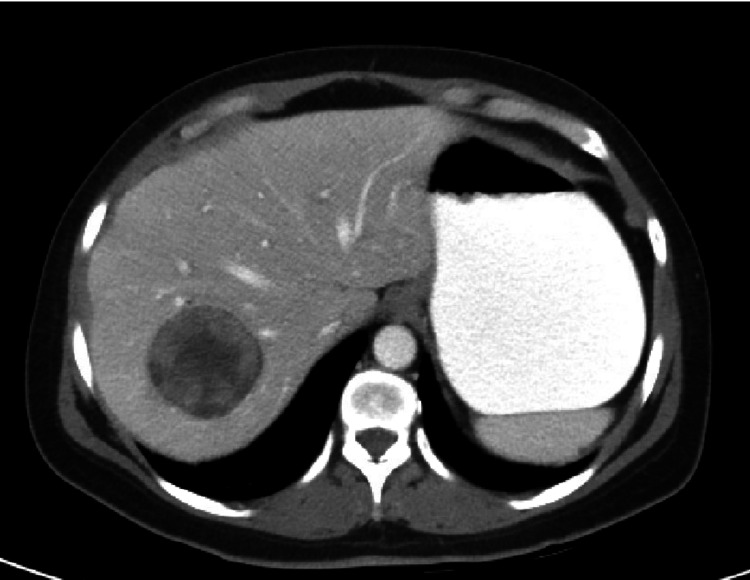
CT abdomen oral and IV contrast axial with portal phase show well-defined, centrally hypodense peripherally enhancing heterogenous density lesion in segment 7 of the liver. Contrast seen in the stomach.

**Figure 2:**
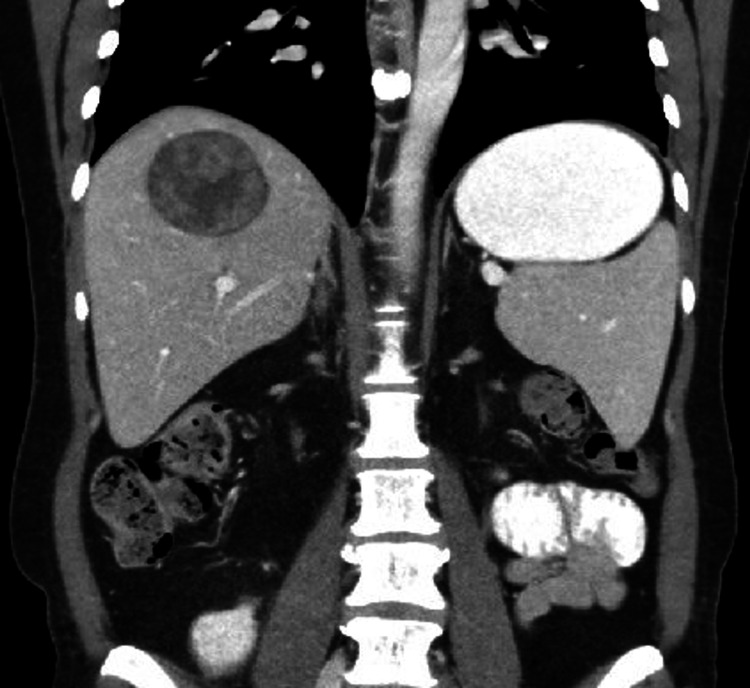
Coronal pane CT demonstrating a well-defined, centrally hypodense mass with peripheral enhancement in segment 7 of the liver.

A 50-year-old female patient presented to the emergency department of a university hospital with dysuria, chills, and pain in the lower right quadrant, as well as general back pain. She had undergone a kidney transplant more than 14 years prior to the current presentation. Her past medical history included hypertension, type 2 diabetes mellitus, hyperlipidemia, recurrent urinary tract infection, left middle cerebral artery (MCA) stroke, and chronic back pain. She was on tacrolimus and prednisone. CT abdomen with oral and IV contrast was obtained and a mass was noted in the liver (Figures 1, 2).

**Figure 3:**
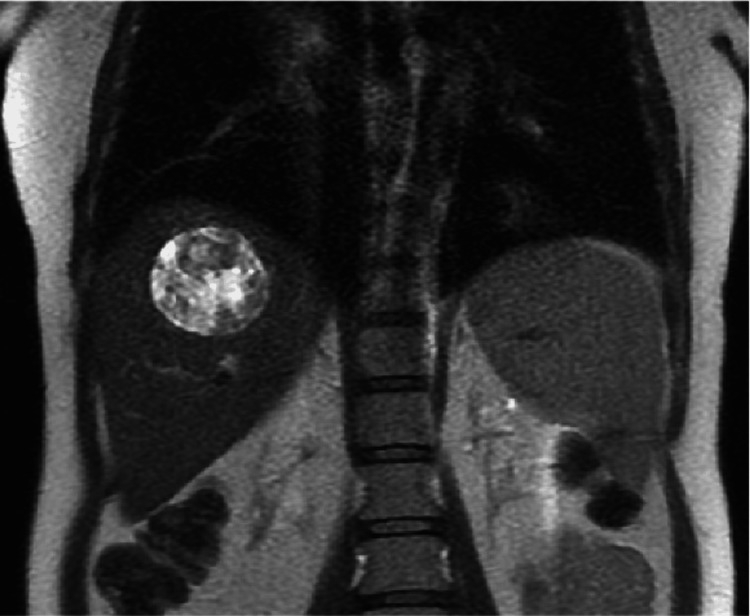
Coronal plane MRI abdomen T2 demonstrating well-circumscribed heterogenous hyperintense mass in segment 7 of the liver.

**Figure 4:**
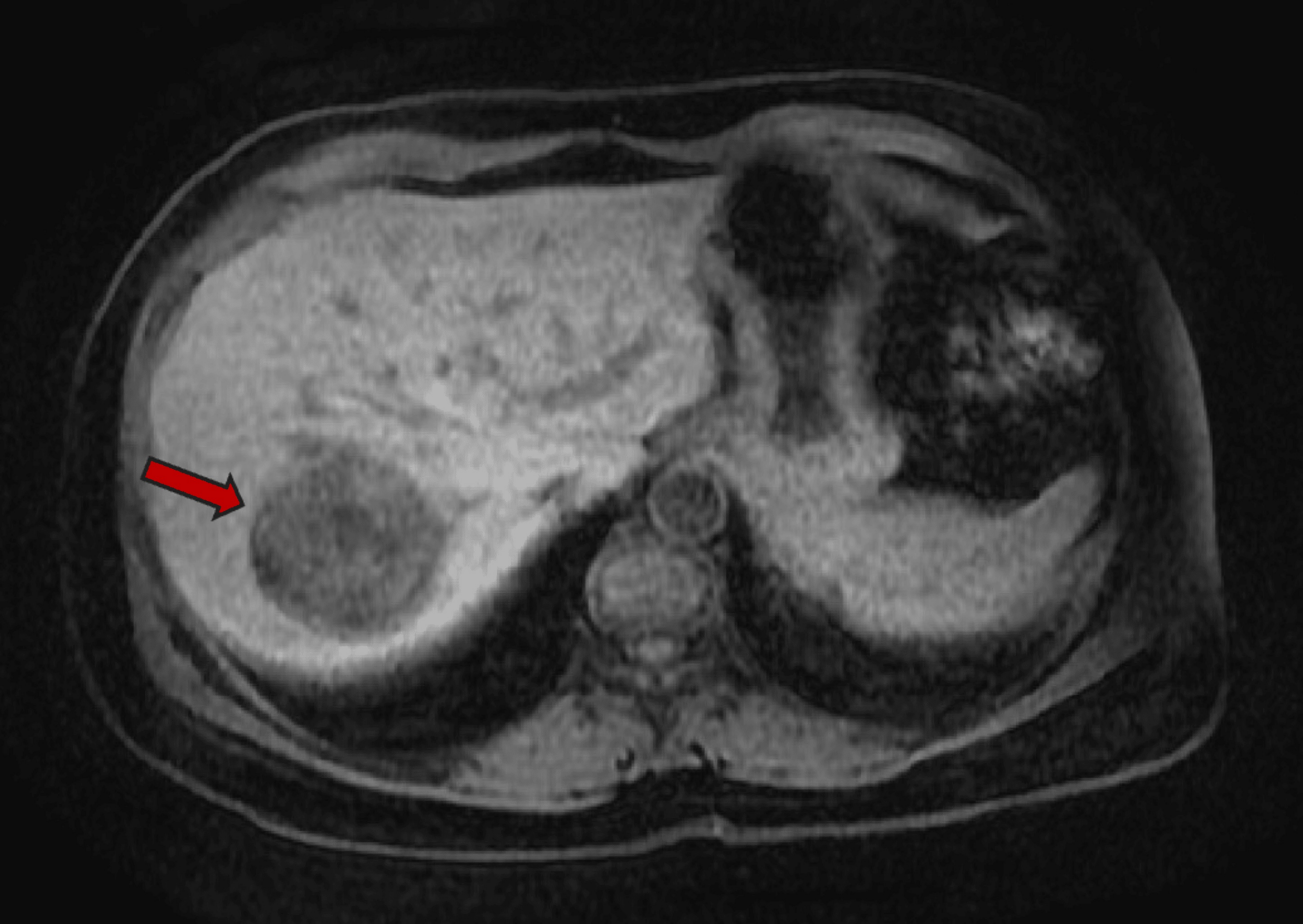
Axial plane MRI abdomen T1 non-contrast study shows central hypointense lesion

**Figure 5:**
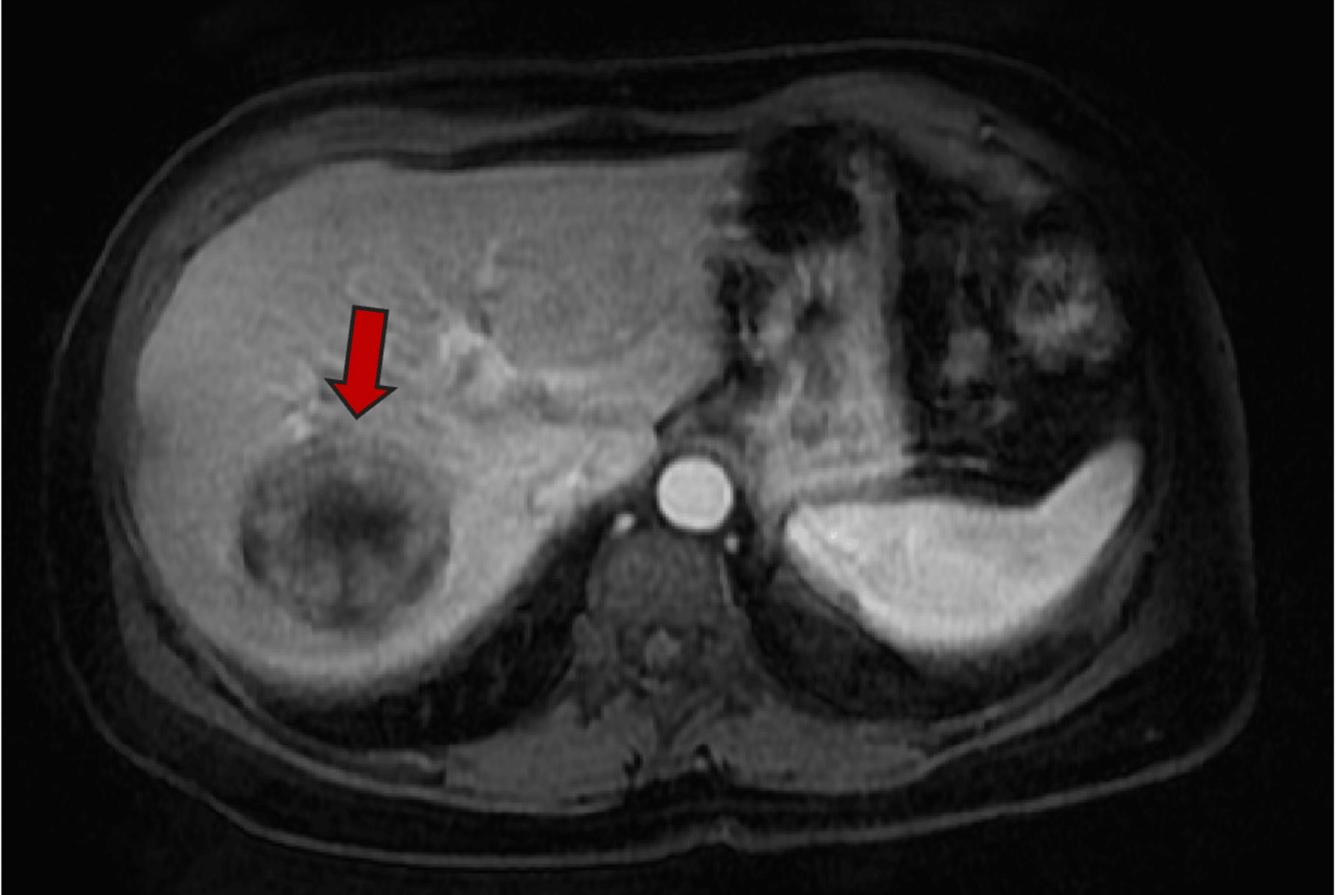
Axial plane MRI abdomen T1 arterial phase shows peripheral enhancement of the segment 7 lesion

**Figure 6:**
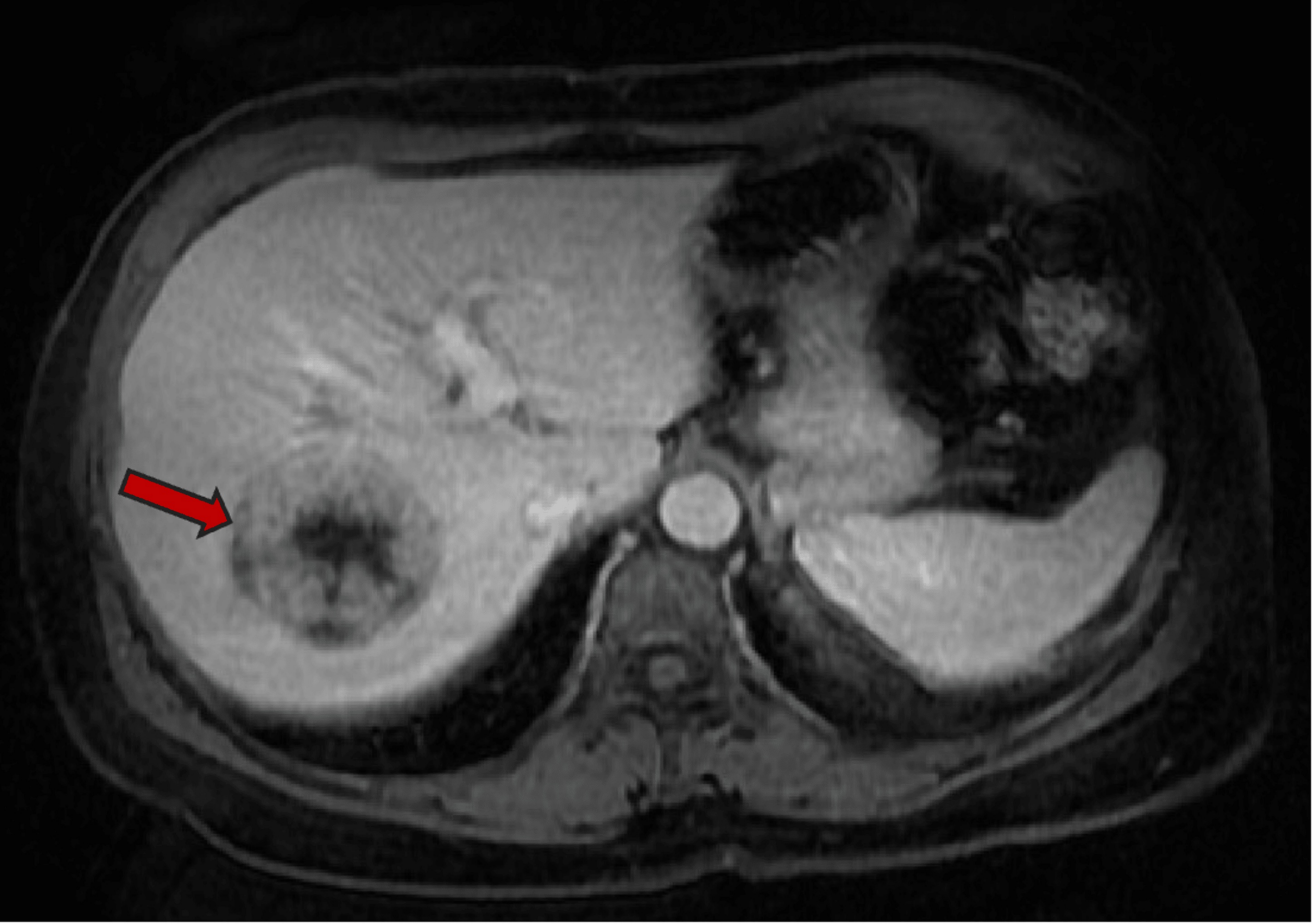
Axial plane MRI abdomen T1 with delayed phase demonstrates progressive enhancement in areas with increased T1 signal. There is no centripetal peripheral nodular enhancement.

**Figure 7:**
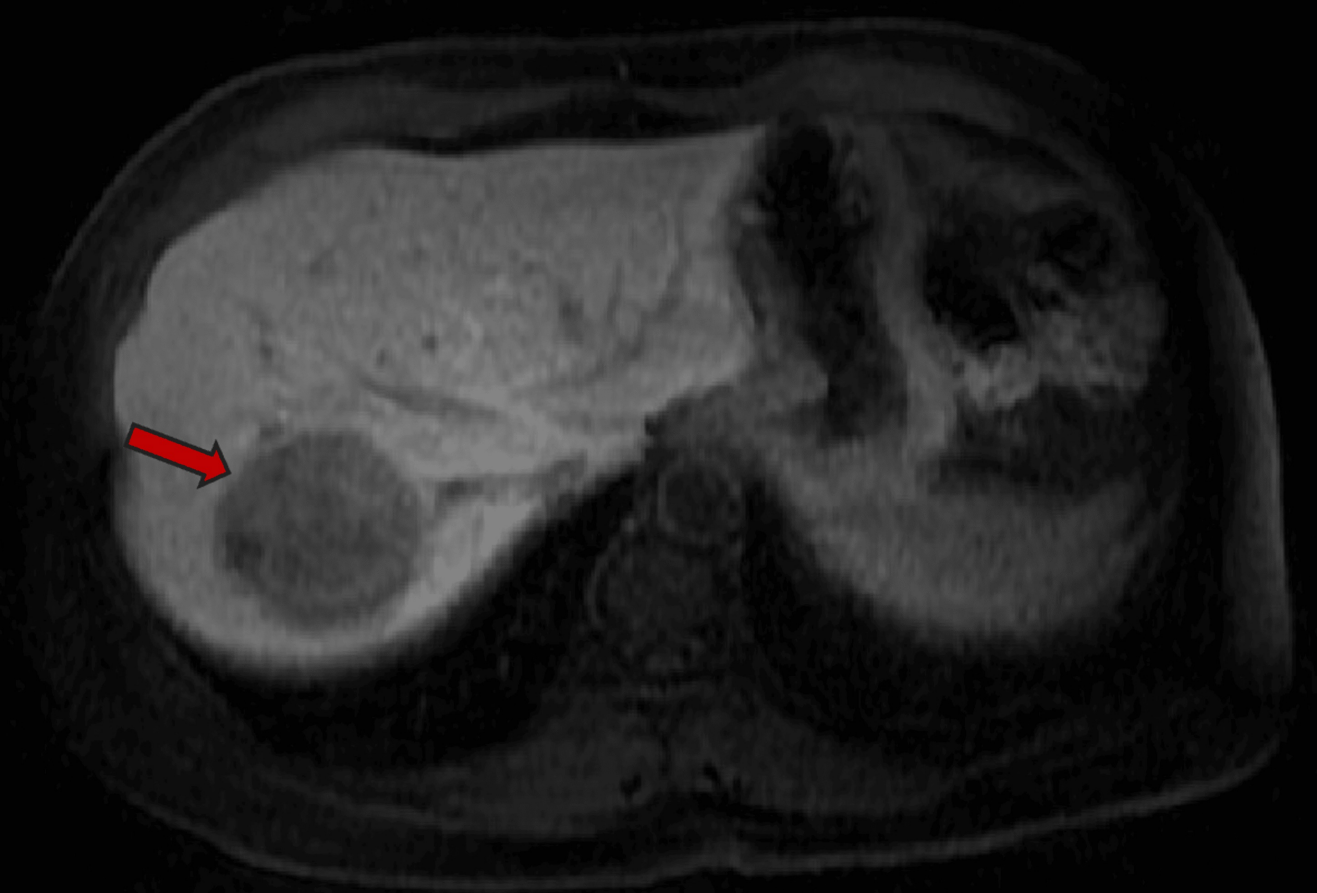
MRI (axial plane) post-contrast hepatobiliary phase (20 minutes) with Eovist demonstrates decreased signal/nonenhancement of the lesion.

The GI team was consulted and recommended an MRI of the liver to better outline the mass and assess whether it could be biopsied (Figures 3-7).

**Figure 8:**
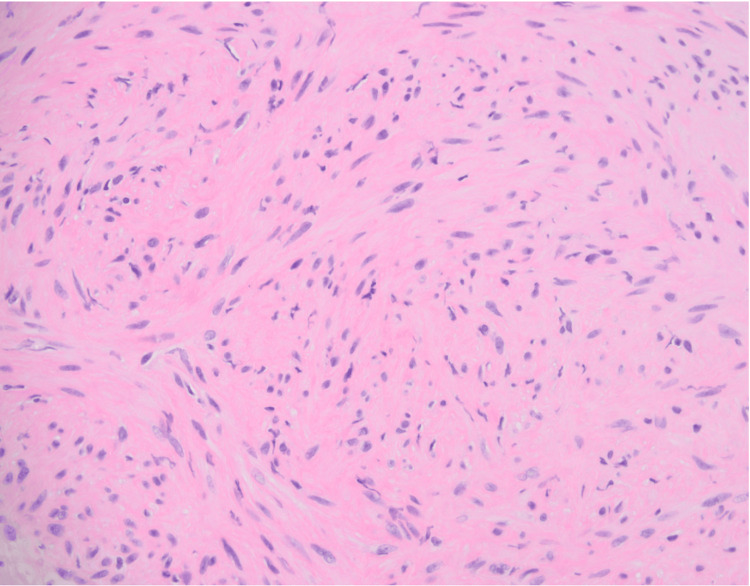
Cells positive for EBV in-situ hybridization and smooth muscle actin. EBV: Epstein Barr virus

Interventional radiology was consulted to perform a biopsy of the mass, which was subsequently determined to be EBV-SMT (Figure 8). A multi-disciplinary team, including Hepatology, Surgery, Oncology, Radiation Oncology, Interventional Radiology, and Pathology, recommended that the patient follow up with her transplant team to determine an appropriate reduction of immunosuppression and monitor for tumor progression.

## Discussion

EBV-SMTs are a rare entity typically occurring in immunocompromised individuals, including those with organ transplantation, HIV/AIDS, and congenital immunodeficiency. In this case, we report a rare instance of EBV-SMT in a patient with a history of kidney transplantation. 

The pathogenesis of EBV-SMT remains unclear, but it is thought to be linked to the reactivation of dormant EBV. More specifically, research suggests that mTOR signaling pathways may play a role in the development and proliferation of EBV-SMT [9]. Other pathways, such as CD21 in HIV-positive patients, have also been proposed [10]. In the case of EBV-SMT, modification of immunosuppression to increase CD4 count resulted in a reduction of EBV-SMT size [10], and it was found that as CD4 levels increased in an HIV-positive male patient, tumor size shrank concurrently [10]. 

The patient presented with symptoms of a urinary tract infection, and a liver lesion was incidentally discovered. Histopathology confirmed the diagnosis of EBV-SMT. Our case offers a unique perspective on EBV-SMT, as most cases in existing literature are reported within the CNS [11]. Notably, EBV-SMT is not commonly associated with mortality [11], which may explain why it is often identified incidentally on imaging. 

Management of EBV-SMTs is complex and often individualized, given the rarity of these tumors and their association with immunosuppression. The primary approach involves surgical resection of the tumor when feasible, as it offers the best chance for symptom relief and disease control. However, recurrence and multifocal disease are common in patients with severe immune dysfunction [7]. For immunocompromised patients, optimizing the immune status, where possible, can reduce the risk of recurrence and prevent the progression of these tumors [7]. In patients who have undergone organ transplantation, reduction of immunosuppressive therapy may sometimes be beneficial, though this must be carefully weighed against the risk of graft rejection [7,12]. For our patient, a multidisciplinary team recommended modifying HIV treatment immunosuppression. 

## Conclusions

The present case adds a unique perspective to the existing literature on EBV-SMT. Traditionally, EBV-SMT is found to occur in the CNS; our case highlights the importance of including EBV-SMT in a differential diagnosis for benign tumors found in immunosuppressed patients. Furthermore, we emphasize that a multidisciplinary approach is needed to ensure both the correct diagnosis and treatment of EBV-SMT. Further credence should be given to balancing the priorities of preventing organ rejection in post-transplant patients and preventing disease occurrence in patients undergoing immune-modulating pharmacotherapy. Future research should further explore the molecular dynamics and optimal treatment of EBV-SMT.
